# Comparison of the predictive performance of three lymph node staging systems for late-onset gastric cancer patients after surgery

**DOI:** 10.3389/fsurg.2024.1376702

**Published:** 2024-06-11

**Authors:** Sheng Chen, Ping’an Ding, Qun Zhao

**Affiliations:** ^1^ Affiliated Hospital of Hebei University, Baoding, Hebei, China; ^2^ The Third Department of Surgery, The Fourth Hospital of Hebei Medical University, Shijiazhuang, Hebei, China; ^3^ Hebei Key Laboratory of Precision Diagnosis and Comprehensive Treatment of Gastric Cancer, Shijiazhuang, Hebei, China; ^4^ Big Data Analysis and Mining Application for Precise Diagnosis and Treatment of Gastric Cancer Hebei Provincial Engineering Research Center, Shijiazhuang, Hebei, China

**Keywords:** XGBoost, late-onset gastric cancer, log odds of positive lymph nodes, lymph node stage, nomogram

## Abstract

**Introduction:**

Lymph node (LN) status is a vital prognostic factor for patients. However, there has been limited focus on predicting the prognosis of patients with late-onset gastric cancer (LOGC). This study aimed to investigate the predictive potential of the log odds of positive lymph nodes (LODDS), lymph node ratio (LNR), and pN stage in assessing the prognosis of patients diagnosed with LOGC.

**Methods:**

The LOGC data were obtained from the Surveillance, Epidemiology, and End Results database. This study evaluated and compared the predictive performance of three LN staging systems. Univariate and multivariate Cox regression analyses were carried out to identify prognostic factors for overall survival (OS). Three machine learning methods, namely, LASSO, XGBoost, and RF analyses, were subsequently used to identify the optimal LN staging system. A nomogram was built to predict the prognosis of patients with LOGC. The efficacy of the model was demonstrated through receiver operating characteristic (ROC) curve analysis and decision curve analysis.

**Results:**

A total of 4,743 patients with >16 removed lymph nodes were ultimately included in this investigation. Three LN staging systems demonstrated significant performance in predicting survival outcomes (*P* < 0.001). The LNR exhibited the most important prognostic ability, as evidenced by the use of three machine learning methods. Utilizing independent factors derived from multivariate Cox regression analysis, a nomogram for OS was constructed.

**Discussion:**

The calibration, C-index, and AUC revealed their excellent predictive performance. The LNR demonstrated a more powerful performance than other LN staging methods in LOGC patients after surgery. Our novel nomogram exhibited superior clinical feasibility and may assist in patient clinical decision-making.

## Introduction

Gastric cancer (GC), a primary global health concern, has become the fifth most diagnosed malignancy and the fourth leading cause of cancer-related mortality worldwide. According to the Global Cancer Statistics, it is estimated that 1,089,103 new GC cases were diagnosed, resulting in 768,793 deaths worldwide in 2020 (1). Projected estimates suggest that by 2040, the number of new cases may increase to 1.77 million, with the number of deaths potentially reaching 1.27 million (2). In recent decades, with the advancement of screening and therapeutic strategies, the incidence and mortality of GC have decreased substantially in most parts of the world, especially in some Western countries (2, 3). However, with the aging of the population, the disease burden on middle-aged and elderly people has increased (4). The number of middle-aged and elderly GC patients who are diagnosed is expected to increase gradually (5). According to research findings, the incidence, mortality and disability-adjusted life-years burden of patients with late-onset gastric cancer (LOGC) are greater than those of patients with early-onset GC in China (6). Therefore, finding a precise, convenient, accurate, and effective risk model is vital for predicting the clinical prognosis of these patients and selecting the optimal treatment.

Lymph node metastasis (LNM) is the site of disease spread in >50% of GC patients and is closely related to early recurrence and poor prognosis (7, 8). The regional lymph node (LN) status is a valid criterion for considering perioperative chemotherapy (9). Postoperative therapy is typically recommended for patients with advanced disease, such as N1 or N2 (10). The number of metastatic LNs in GC patients is a great indicator of prognosis and recurrence (11). Accurate LN staging plays a critical role in the selection of treatment strategies and the determination of prognosis for LOGC patients after surgery. Currently, the N-stage classification scheme of the tumor–node–metastasis (TNM) system is widely recommended for classifying LN status (12). However, due to some clinical limitations, the use of the pN staging system, such as stage migration, has been disputed by some researchers (13, 14), which may cause the misorientation of treatment selection and the inaccuracy of prognosis prediction (15). In the last decade, several LN classification factors, including the lymph node ratio (LNR) and log odds ratio of positive lymph nodes (LODDS), have been applied to illustrate LN status as a substitution for the pN staging system. The LNR and LODDS have been demonstrated to be prognostic markers for numerous malignancies, such as lung carcinoma (16), esophageal carcinoma (17), breast cancer (18), rectal cancer (19, 20), and GC (21, 22). However, there are some controversies in which the nodal staging system is the most applicable for evaluating the accuracy of LN status, and there is insufficient evidence for screening the most appropriate nodal staging system for LOGC patients. Therefore, it is essential to explore a more efficacious and accurate LN scheme for LOGC patients to improve prognosis and guide therapeutic strategy decisions.

Thus, this study aimed to assess the appropriate nodal staging system by comparing the predictive prognostic ability of the pN, LNR, and LODDS nodal staging systems among the LOGC group and utilized the most optimal scheme to construct a nomogram model for predicting survival in cases with LOGC after surgery.

## Materials and methods

### Database and population selection

The data utilized in this study were collected from the Incidence—SEER 17 Registries Research Data, November 2022 Sub (2000–2020) dataset in the SEER database (available at https://seer.cancer.gov/). Clinical data of GC patients selected from 2010 to 2020 were downloaded from the SEER*Stat software (version 8.4.2).

The age threshold of the LOGC did not coincide with the studies. Most scholars have defined the age limits for the LOGC and EOGC as above 40 or 50 years, respectively (23–25), while some scholars have used 55 or 60 years as the cutoff age (26, 27). According to the previous studies, 50 years was used as the dividing variable in our study.

**Figure 1:**
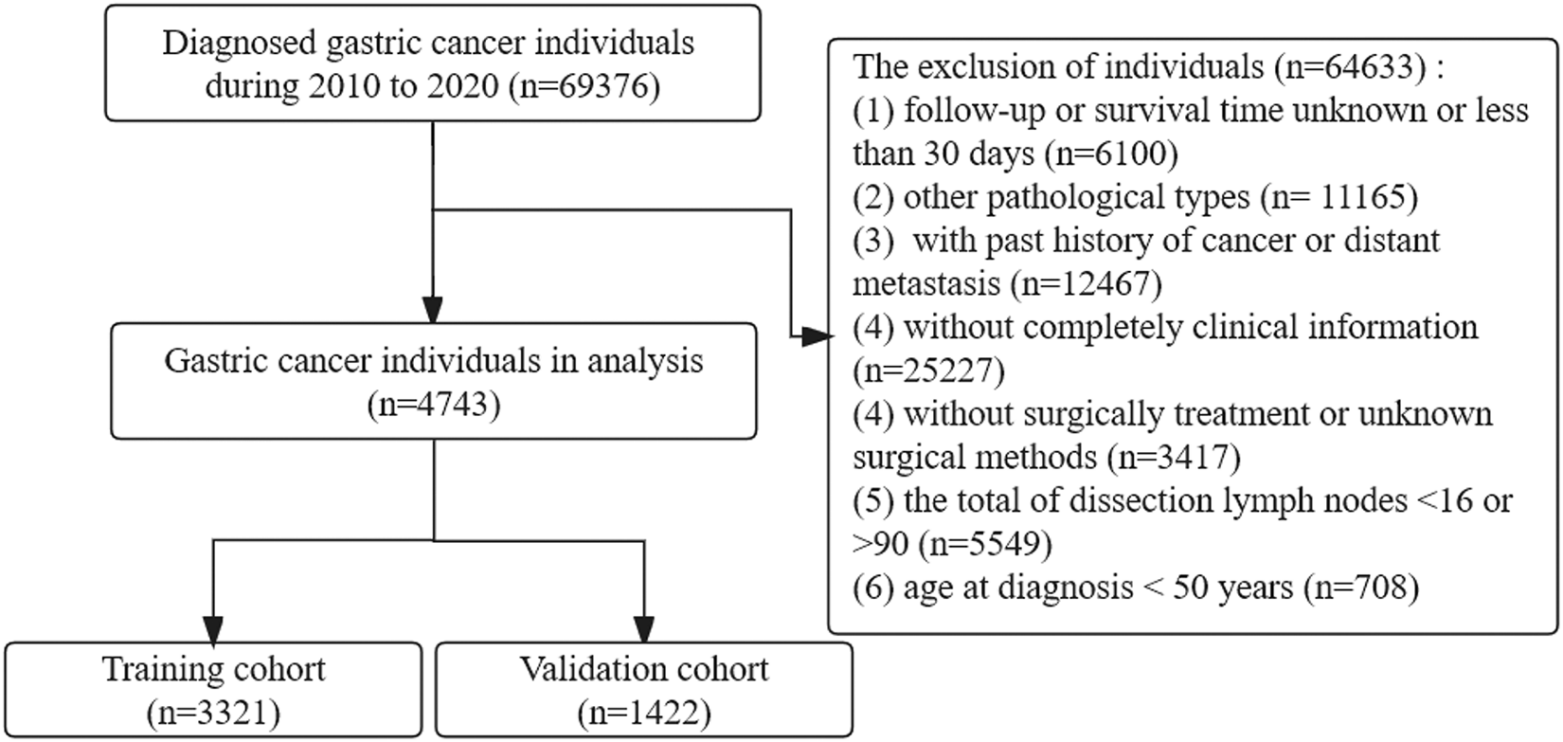
Flowchart of selecting the individuals with LOGC in this study.

Eligible patients were selected according to the following criteria: patients diagnosed with GC confirmed by pathology (ICD-O-3 code: histological type recodes 8010–8231 and 8255–8576 and tumor site recodes C16.0–C16.6 and C16.8–C16.9). The patient exclusion criteria were the following: (1) patients with a history of cancer or distant metastasis, (2) who are under the age of 50 years at diagnosis, (3) without surgical treatment, (4) without complete clinical information (such as gender, diagnosis age, tumor size, TNM stage, and surgery recode), (5) who are involving other pathological types, (6) with a total score of dissection LNs of <16, and (7) with a follow-up or survival time of <30 days. The flowchart of the study selection process is depicted in Figure 1. Subsequently, 4,743 LOGC patients were selected for the subsequent analyses. The data were randomly divided into a training set (*n* = 3,321) and a validation set (*n* = 1,422) via the “caret” R package at a ratio of 7:3.

### Collections of variables and outcomes

Several demographic characteristics, such as age, sex, race, and marital status, were included. The clinical variables such as T stage, N stage, histological type, tumor grade, tumor size, number of removed LNs, number of positive LNs, follow-up time, and follow-up status were extracted from the SEER database. The LNR was calculated by dividing the number of metastatic LNs by the total number of nodes examined. The LODDS was calculated by the following formula from previous studies (28): log [(the total number of removed LNs +0.05)/(the number of metastatic LNs +0.05)]. According to the ICD-O-3 codes, the cases were divided into three types: intestinal type (8140, 8144, 8210–8211, 8260, and 8480–8481), diffuse type (8020–8022, 8142, 8145, and 8490), and other. For primary tumor sites, C16.0 recodes were allotted into the cardia, while C16.1–C16.2 and C16.5–C16.6 recoded into the middle site, C16.3–C16.4 into the distal site, and C16.8–C16.9 into the other site. The optimal cutoff values for successive LNR and LODDS were defined by X-Tile software. LNR was divided into <0.05, 0.05–0.43, and >0.43, while LODDS was divided into less than −4.26, −4.26–1.23, and greater than −1.23.

The endpoint was overall survival (OS). The OS was obtained from the “vital status recode” column in the SEER dataset.

### Prognosis-related feature screening

In the framework of this investigation, a univariate Cox regression analysis was conducted to scrutinize the influence of clinical factors on survival duration within the training cohort via the “survival” package in R software. The dataset comprised 13 subjects, including age, sex, race, marital status, T stage, N stage, tumor stage, successive LNR and LODDS, classification LNR and LODDS, primary tumor site, tumor size, histologic grade, and chemotherapy. Those with statistically significant features in the univariate Cox analysis were imported into the multivariate Cox regression analysis to further screen prognosis-related features.

### The optimal LN system selection

For optimal LN system selection, first, the ability of three LN staging systems to predict patient prognosis was compared via the C-index, Akaike information criterion (AIC), and Bayesian information criterion (BIC). Furthermore, receiver operating characteristic (ROC) curves and areas under the curve (AUCs) were generated to evaluate the predictive value of the three LN systems.

Finally, three machine learning algorithms, namely, least absolute shrinkage (LASSO) regression (29), random forest (30), and Extreme Gradient Boosting (31) (XGBoost), were applied to various dimensions. All three methods involve direct selection of the original features without any linear combination or transformation, and the selected features are consistent with the original features. It can calculate feature importance scores, allowing users to understand the contribution of each feature to the model's prediction. This insight can be valuable for feature selection and model interpretation. The LASSOCV module of the “glmnet” R package was utilized to conduct LASSO regression (32). XGBoost analysis was used to extract and analyze the importance of each feature with the “XGBoost” R package (33). The random forest classifier was trained on the training cohort generated by the “randomForestSRC” R package (34). The feature importance was extracted via the function of “feature importance”.

### Construction of a nomogram

A nomogram is a graphical representation of mathematical relationships and is usually used to estimate the results of a formula via graphical means. In this study, a nomogram for OS was created to estimate the prognosis of LOGC patients based on the optimal LN system. The calibration plot, concordance index, ROC curve, and decision curve analysis were plotted to evaluate the accuracy of the prognostic nomogram in both the training and validation cohorts.

### Statistical analysis

R software (version 4.3.1) and IBM SPSS (version 26) were utilized to perform all analyses. A *P*-value of <0.05 was considered to indicate statistical significance. The *χ*^2^ test and *t*-test were used to compare the relationships between the categorical variables, and the means and medians were calculated to present the descriptive variables. A *P*-value of <0.05 was considered to indicate statistical significance. The dummy variables were applied to account for the multinomial variable data via one-hot encoding. The ordinal and interval variable data were substituted for numeric variables. The TNM stages of the patients were redetermined based on the eighth edition TNM staging system of the AJCC.

## Results

### Patient demographic and clinical characteristics

A total of 4,743 LOGC participants were included in this study and were randomly classified into the training group (*n* = 3,321) and the validation group (*n* = 1,422) via the “caret” package in R software at a ratio of 7:3. All the participants aged >50 years at the time of the initial diagnosis, with a median age of 68.5 (SD 9.84) years, while the median ages in the training and validation groups were 65.9 (SD 9.86) and 65.6 (SD 9.8) years, respectively. Interestingly, most patients were diagnosed at 60–69 years of age. In addition, the number of dissected LNs in each patient was no less than 16, and the median was 22.4 (SD 12.2). The median LNR was 0.187 (SD 0.261), while the median LODDS was −3.15 (SD 2.94). There were no significant differences between the two groups regarding any of the clinical factors, and further details are presented in Table 1.

**Table 1:** Baseline demographic and clinicopathological features of the patients.

Variable	Total	Training cohort	Validation cohort	*P*
	(*N* = 4,743)	(*N* = 3,321)	(*N* = 1,422)	
Age (years)				
Mean (SD)	65.8 (9.84)	65.9 (9.86)	65.6 (9.80)	0.279
Gender				
Female	1,741 (36.7%)	1,193 (35.9%)	548 (38.5%)	0.093
Male	3,002 (63.3%)	2,128 (64.1%)	874 (61.5%)	
Primary tumor site				
Distal site	1,411 (29.7%)	979 (29.5%)	432 (30.4%)	0.075
Cardia	1,122 (23.7%)	820 (24.7%)	302 (21.2%)	
Middle site	1,537 (32.4%)	1,054 (31.7%)	483 (34.0%)	
Other	673 (14.2%)	468 (14.1%)	205 (14.4%)	
Year at diagnosis				
2010–2013	1,981 (41.8%)	1,362 (41.0%)	619 (43.5%)	0.174
2014–2017	2,008 (42.3%)	1,414 (42.6%)	594 (41.8%)	
2018–2020	754 (15.9%)	545 (16.4%)	209 (14.7%)	
Tumor size				
Mean (SD)	49.0 (42.5)	48.5 (40.4)	49.9 (47.0)	0.788
<2 cm	869 (18.3%)	584 (17.6%)	285 (20.0%)	0.120
>5 cm	1,522 (32.1%)	1,082 (32.6%)	440 (31.0%)	
2–5 cm	2,352 (49.6%)	1,655 (49.8%)	697 (49.0%)	
LNR				
Mean (SD)	0.187 (0.261)	0.185 (0.260)	0.191 (0.263)	0.312
<0.05	2,302 (48.5%)	1,628 (49.0%)	674 (47.4%)	0.413
0.05–0.43	1,635 (34.5%)	1,143 (34.4%)	492 (34.6%)	
>0.43	806 (17.0%)	550 (16.6%)	256 (18.0%)	
LODDS				
Mean (SD)	−3.15 (2.94)	−3.18 (2.94)	−3.08 (2.92)	0.299
Less than −4.26	1,967 (41.5%)	1,397 (42.1%)	570 (40.0%)	0.388
−4.26 to −1.23	1,367 (28.8%)	941 (28.3%)	426 (30.0%)	
Greater than −1.23	1,409 (29.7%)	983 (29.6%)	426 (30.0%)	
Marital status				
Married	3,011 (63.5%)	2,108 (63.5%)	903 (63.5%)	0.833
Unmarried	1,547 (32.6%)	1,087 (32.7%)	460 (32.3%)	
Unknown	185 (3.9%)	126 (3.8%)	59 (4.2%)	
Radiation				
No/unknown	3,251 (68.5%)	2,282 (68.7%)	969 (68.1%)	0.724
Yes	1,492 (31.5%)	1,039 (31.3%)	453 (31.9%)	
Chemotherapy				
No/unknown	2,011 (42.4%)	1,401 (42.2%)	610 (42.9%)	0.673
Yes	2,732 (57.6%)	1,920 (57.8%)	812 (57.1%)	
Grade				
Well/moderately differentiated	1,533 (32.3)	1,076 (32.4%)	457 (32.1%)	0.886
Poorly/undifferentiated	3,210 (67.7)	2,245 (67.6%)	965 (67.9%)	
Stage				
I	1,219 (25.7%)	860 (25.9%)	359 (25.2%)	0.435
II	1,185 (25.0%)	843 (25.4%)	342 (24.1%)	
III	2,339 (49.3%)	1,618 (48.7%)	721 (50.7%)	
T				
T1	1,104 (23.3%)	772 (23.2%)	332 (23.3%)	0.833
T2	609 (12.8%)	430 (13.0%)	179 (12.6%)	
T3	1,868 (39.4%)	1,317 (39.7%)	551 (38.8%)	
T4	1,162 (24.5%)	802 (24.1%)	360 (25.3%)	
N				
N0	1,745 (36.8%)	1,233 (37.1%)	512 (36.0%)	0.73
N1	914 (19.3%)	637 (19.2%)	277 (19.5%)	
N2	826 (17.4%)	584 (17.6%)	242 (17.0%)	
N3	1,258 (26.5%)	867 (26.1%)	391 (27.5%)	
Histological type				
Diffuse type	1,281 (27.0%)	897 (27.0%)	384 (27.0%)	0.931
Intestinal type	3,186 (67.2%)	2,228 (67.1%)	958 (67.4%)	
Other	276 (5.8%)	196 (5.9%)	80 (5.6%)	
Survival time (month)				
Mean (SD)	42.5 (34.8)	42.3 (34.5)	42.9 (35.3)	0.811
Status				
Alive	2,395 (50.5%)	1,677 (50.5%)	718 (50.5%)	1
Dead	2,348 (49.5%)	1,644 (49.5%)	704 (49.5%)	

### Identification of prognosis-related clinical factors for OS

The results of univariate Cox regression revealed significant associations between survival time and certain clinical variables, including marital status, T stage, N stage, tumor stage, histological type, age at diagnosis, successive LNR and LODDS, and classification of LNR and LODDS. The estimated regression coefficients and hazard ratios (HRs) for each variable are presented in Table 2. Notably, successive LNR and LODDS exhibited statistically significant HRs of 10.32 (95% CI: 8.82–12.07, *p* < 0.05] and 1.28 (95% CI: 1.26–1.30, *p* < 0.05], respectively, indicating that both parameters were related to prognosis. Cox multivariate regression analyses were performed to further determine the associations between pN stage, LODDS, and LNR and OS in LOGC patients. The results showed that the LODDS, LNR, and pN status significantly impacted OS in the LOGC patients (Table 3).

**Table 2:** Univariate analysis of overall survival in the training cohort.

Variable	Univariate	
	HR (95% CI)	*P*
Age (years)		
50–59	Reference	
60–69	1.05 (0.91–1.20)	0.51
70–79	1.18 (1.03–1.36)	0.02
80+	1.76 (1.51–2.05)	<0.05
Gender		
Female	Reference	
Male	1.08 (0.97–1.20)	0.14
Tumor size		
<2 cm	Reference	
2–5 cm	1.02 (0.89–1.17)	0.8
>5 cm	1.39 (1.20–1.61)	<0.05
T stage		
T1	Reference	
T2	1.73 (1.39–2.14)	<0.05
T3	3.07 (2.61–3.62)	<0.05
T4	4.99 (4.21–5.92)	<0.05
Tumor stage		
Stage I	Reference	
Stage II	2.39 (1.99–2.87)	<0.05
Stage III	5.43 (4.61–6.39)	<0.05
Grade		
Well/moderately differentiated	Reference	
Poorly/undifferentiated	1.43 (1.28–1.59)	<0.05
Marital status		
Married	Reference	
Unmarried	1.21 (1.09–1.34)	<0.05
LNR		
<0.05	Reference	
0.05–0.43	2.63 (2.35–2.96)	<0.05
>0.43	5.99 (5.26–6.83)	<0.05
LODDS		
Less than −4.26	Reference	
−4.26 to −1.23	2.18 (1.91–2.49)	<0.05
Greater than −1.23	4.97 (4.40–5.62)	<0.05
N		
N0	Reference	
N1	1.85 (1.58–2.17)	<0.05
N2	2.90 (2.50–3.36)	<0.05
N3	5.13 (4.49–5.86)	<0.05
Race		
White	Reference	
Black	1.04 (0.90–1.20)	0.62
Other/unknown	0.75 (0.67–0.84)	<0.05
Primary tumor site		
Distal site	Reference	
Cardia	1.00 (0.88–1.14)	0.95
Middle	0.91 (0.81–1.03)	0.15
Other	1.03 (0.88–1.20)	0.74
Histological type		
Diffuse type	Reference	
Intestinal	0.79 (0.71–0.88)	<0.05
Other	0.96 (0.78–1.19)	0.72

**Table 3:** Association of pN stage, LNR, and LODDS with OS in the training cohort.

Variable	Multivariate analysis		Multivariate analysis		Multivariate analysis	
	HR (95% CI)	*P*	HR (95% CI)	*P*	HR (95% CI)	*P*
T						
T1	Reference					
T2	1.42 (1.14–1.77)	<0.05	1.46 (1.17–1.82)	<0.05	1.46 (1.17–1.82)	<0.05
T3	2.06 (1.72–2.47)	<0.05	2.17 (1.82–2.59)	<0.05	2.23 (1.87–2.65)	<0.05
T4	2.61 (2.15–3.17)	<0.05	2.62 (2.17–3.17)	<0.05	2.70 (2.23–3.26)	<0.05
Marital status						
Married	Reference					
Unmarried	1.15 (1.04–1.28)	<0.05	1.15 (1.04–1.28)	<0.05	1.17 (1.05–1.29)	<0.05
Age (years)						
50–59	Reference					
60–69	1.12 (0.97–1.28)	0.11	1.12 (0.98–1.29)	0.11	1.09 (0.95–1.25)	0.21
70–79	1.39 (1.21–1.59)	<0.05	1.40 (1.22–1.61)	<0.05	1.37 (1.19–1.57)	<0.05
80+	1.91 (1.63–2.24)	<0.05	1.87 (1.60–2.19)	<0.05	1.78 (1.52–2.09)	<0.05
Histological type						
Diffuse	Reference					
Intestinal	0.93 (0.82–1.05)	0.22	0.93 (0.82–1.04)	0.21	0.94 (0.93–1.06)	0.31
Other	0.97 (0.79–1.21)	0.81	0.96 (0.78–1.19)	0.70	0.97 (0.79–1.20)	0.80
Tumor size						
<2 cm	Reference					
2–5 cm	0.88 (0.76–1.01)	0.08	0.88 (0.75–1.02)	0.08	0.90 (0.78–1.05)	0.17
>5 cm	0.89 (0.77–1.04)	0.14	0.84 (0.73–0.97)	0.02	0.90 (0.78–1.05)	0.17
Grade						
Poorly/undifferentiated	Reference					
Well/moderately						
Differentiated	1.04 (0.92–1.17)	0.56	1.04 (0.92–1.17)	0.54	1.06 (0.94–1.19)	0.38
N						
N0	Reference					
N1	1.55 (1.31–1.83)	<0.05				
N2	2.24 (1.91–2.63)	<0.05				
N3	3.52 (3.02–4.10)	<0.05				
LODDS						
Less than −4.26			Reference			
−4.26 to −1.23			3.56 (3.10–4.09)	<0.05		
Greater than −1.23			1.81 (1.58–2.08)	<0.05		
LNR						
<0.05					Reference	
0.05–0.43					2.13 (1.89–2.41	<0.05
>0.43					4.16 (3.60–4.83)	<0.05

#### Selection of the optimal LN system

**Figure 2:**
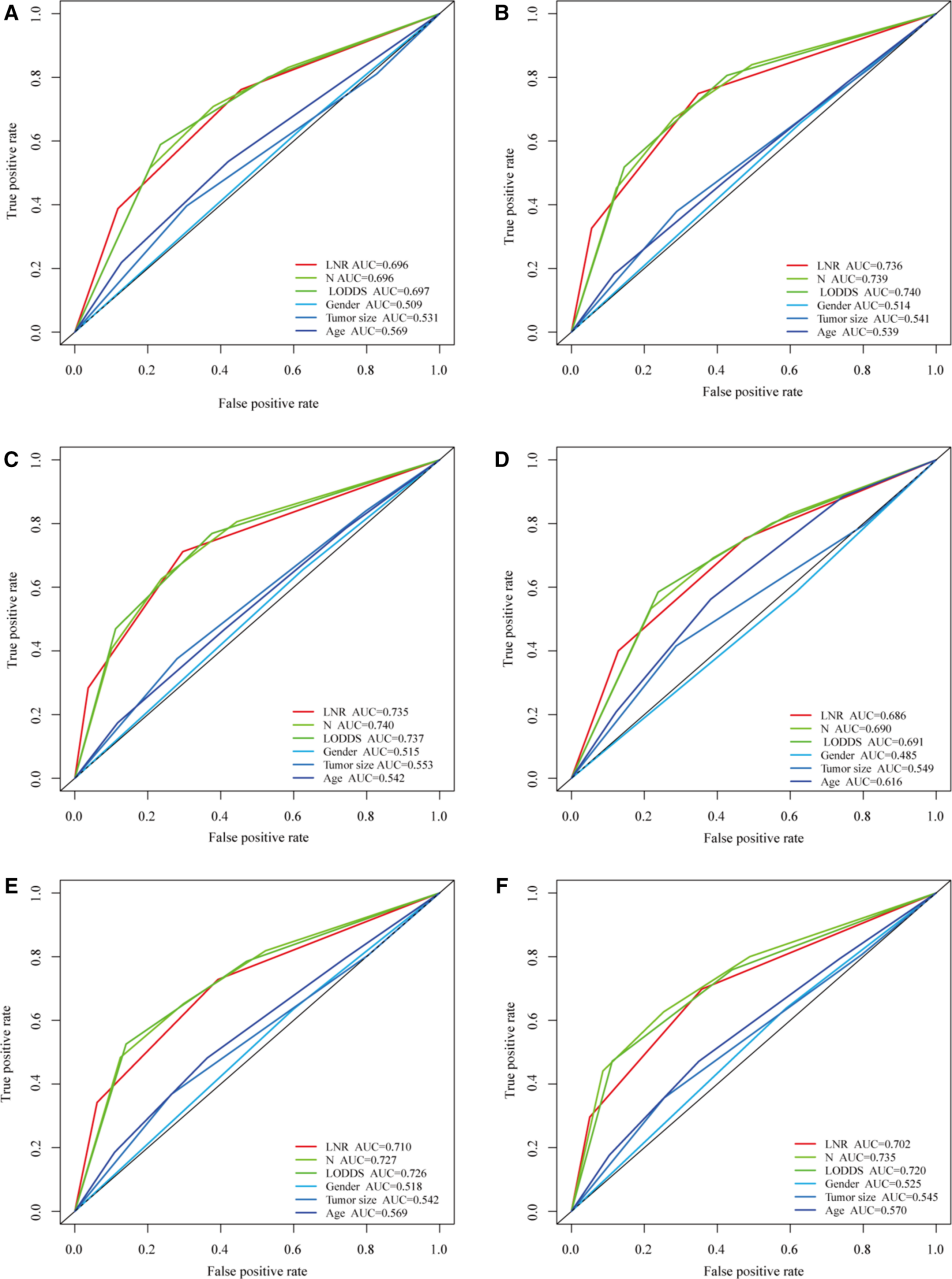
Comparison of the three LN systems in the training and validation cohorts. (**A–C**) ROC curves for predicting 1-, 3-, and 5-year overall survival (OS) in the training cohort. (**D–F**) ROC curves for predicting 1-, 3-, and 5-year OS in the validation cohort.

The predictive prognostic capability of the three LN systems was similar in the training and validation cohorts (Table 4). In the training cohort, the C-indexes for the LNR, LODDS, and pN were 0.679, 0.682, and 0.681, respectively, while in the validation cohort, the C-indexes were 0.664, 0.672, and 0.676, respectively. In addition, the AIC values of each system were 24,112.03, 24,133.89, and 24,174.96 in the training cohort and 9,227.21, 9,217.83, and 9,207.59 in the validation cohort, respectively. To further explore the ability of the nomogram to predict patient prognosis, the area under the curve (AUC) curves were plotted, as shown in Figure 2. In the training cohort, the time-dependent 1-year AUC values for the LNR, LODDS, and pN were 0.696 (95% CI: 0.672–0.720), 0.697 (95% CI: 0.673–0.721), and 0.696 (95% CI: 0.672–0.720), respectively. The 3-year AUC values were 0.736 (95% CI: 0.718–0.753), 0.740 (95% CI: 0.723–0.756), and 0.739 (95% CI: 0.721–0.757), and the 5-year AUC values were 0.735 (95% CI: 0.717–0.753), 0.737 (95% CI: 0.718–0.756), and 0.740 (95% CI: 0.720–0.759), respectively. These findings suggested that the differences in the discrimination qualities of the three systems were less significant, which aligned with the aforementioned conclusions. For this reason, the machine learning methods LASSO, XGBoost, and RF were used to further identify the optimal LN system in terms of predictive ability. The following variables were incorporated into this analysis: successive LNR and LODDS, pN, marital status, tumor grade, histological type, age, primary tumor site, tumor size, clinical stage, and sex.

**Table 4:** Prediction performance of the three lymph nodal staging systems.

Variable	C-index (95% CI)	AIC	BIC
Training cohort			
LNR	0.679 (0.667–0.691)	24,112.03	24,122.8
LODDS	0.682 (0.670–0.694)	24,133.89	24,144.70
pN	0.679 (0.667–0.691)	24,174.96	24,191.2
Validation cohort			
LNR	0.664 (0.468–0.860)	9,227.21	9,236.32
LODDS	0.672 (0.476–0.868)	9,217.83	9,226.95
pN	0.676 (0.480–0.872)	9,207.59	9,221.26

**Figure 3:**
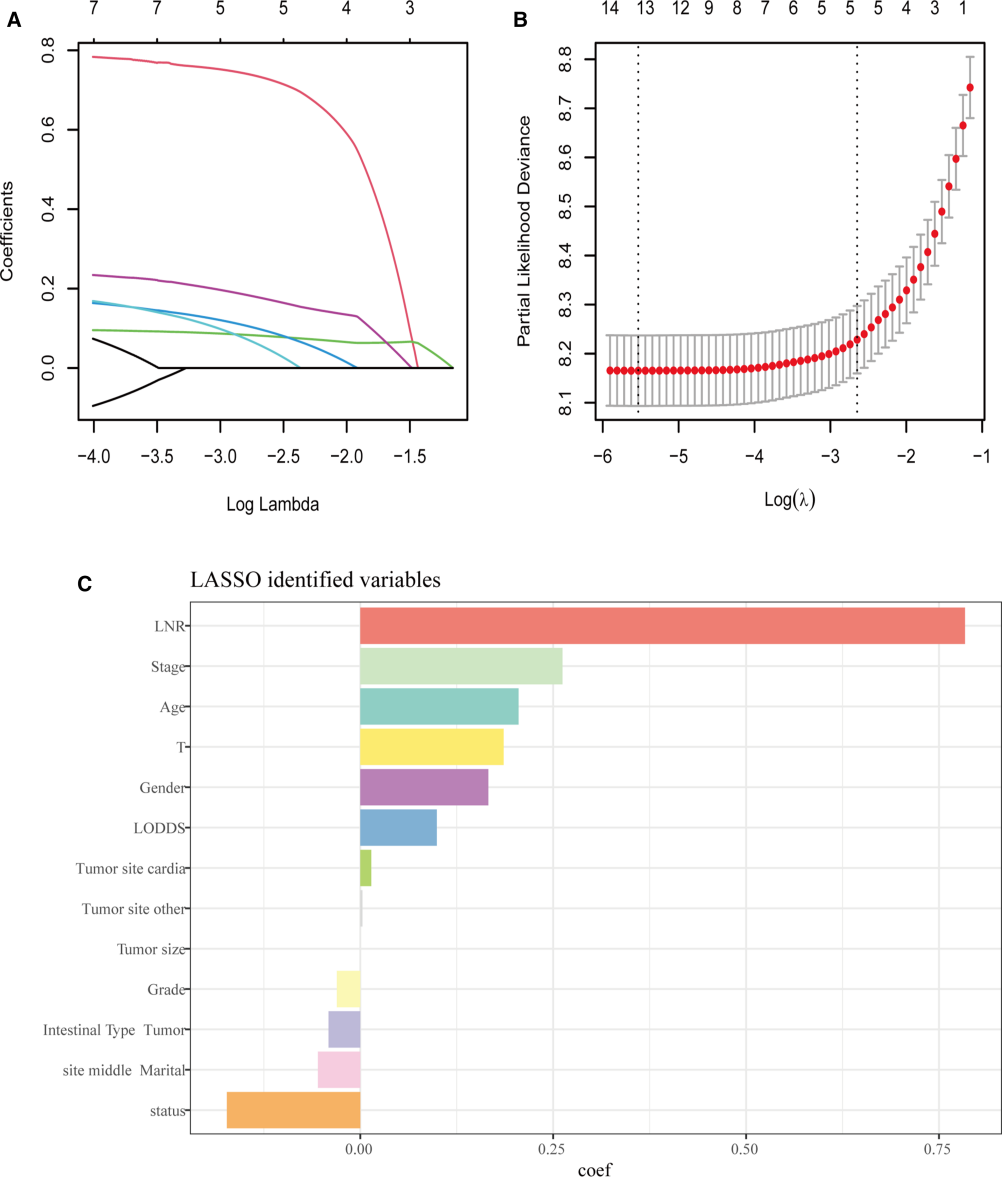
LASSO regression analysis. (**A,B**) LASSO regression to identify the optimal variable. (**C**) The coefficients of each variable in LASSO analysis.

**Figure 4:**
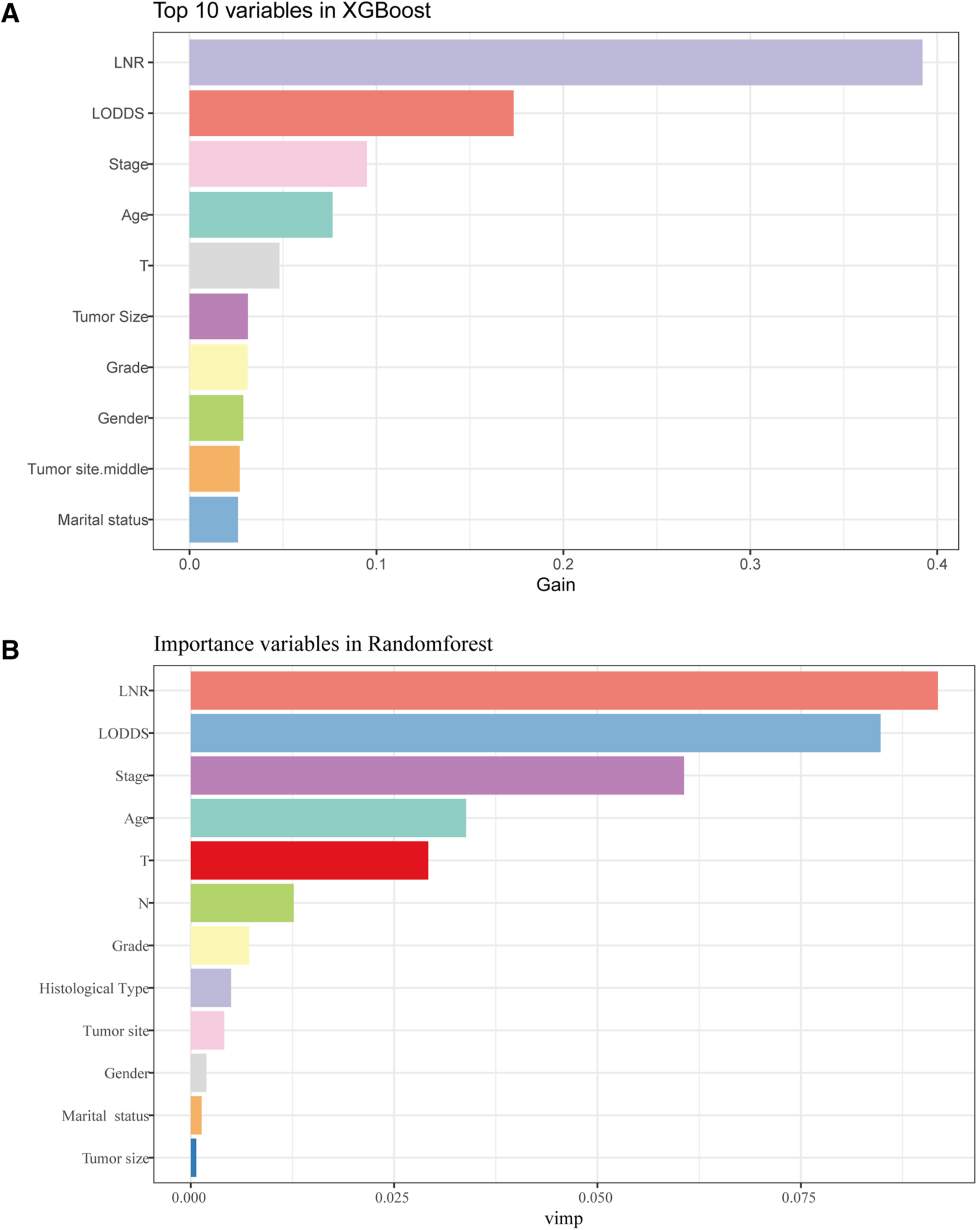
The results of XGBoost and RF analyses. (**A**) The feature importance in XGBoost analysis. (**B**) the importance score of features in RF analysis.

For the LASSO regression analysis, after 10-fold cross-validation and adjustment of the optimal *α* parameter value (*α* = 0.003) to control the strength of regularization, features with 0 values of the coefficient parameter variables were excluded (Figures 3A,B). Then, the importance of features was determined by the absolute values of the coefficients obtained from the final output Lasso model in the training cohort (Figure 3C). The importance of features in a Lasso model can be inferred from the magnitude of the coefficients. We found that the coefficient of the LNR was the largest, which may indicate that the LNR is one of the most important features. Subsequently, XGBoost was performed on the training dataset. The importance values for each variable are shown in Figure 4A. LNR showed the highest importance. The significance of each feature from the random forest analysis is shown in Figure 4B. The finding that the LNR system was the most relevant, and influential feature for prediction was consistent with the abovementioned findings.

Based on the results of three machine learning methods, the LNR system was selected in this study as the optimal system for evaluating the status of LNs in patients with LOGC.

### Development and validation of a nomogram based on the LNR

**Figure 5:**
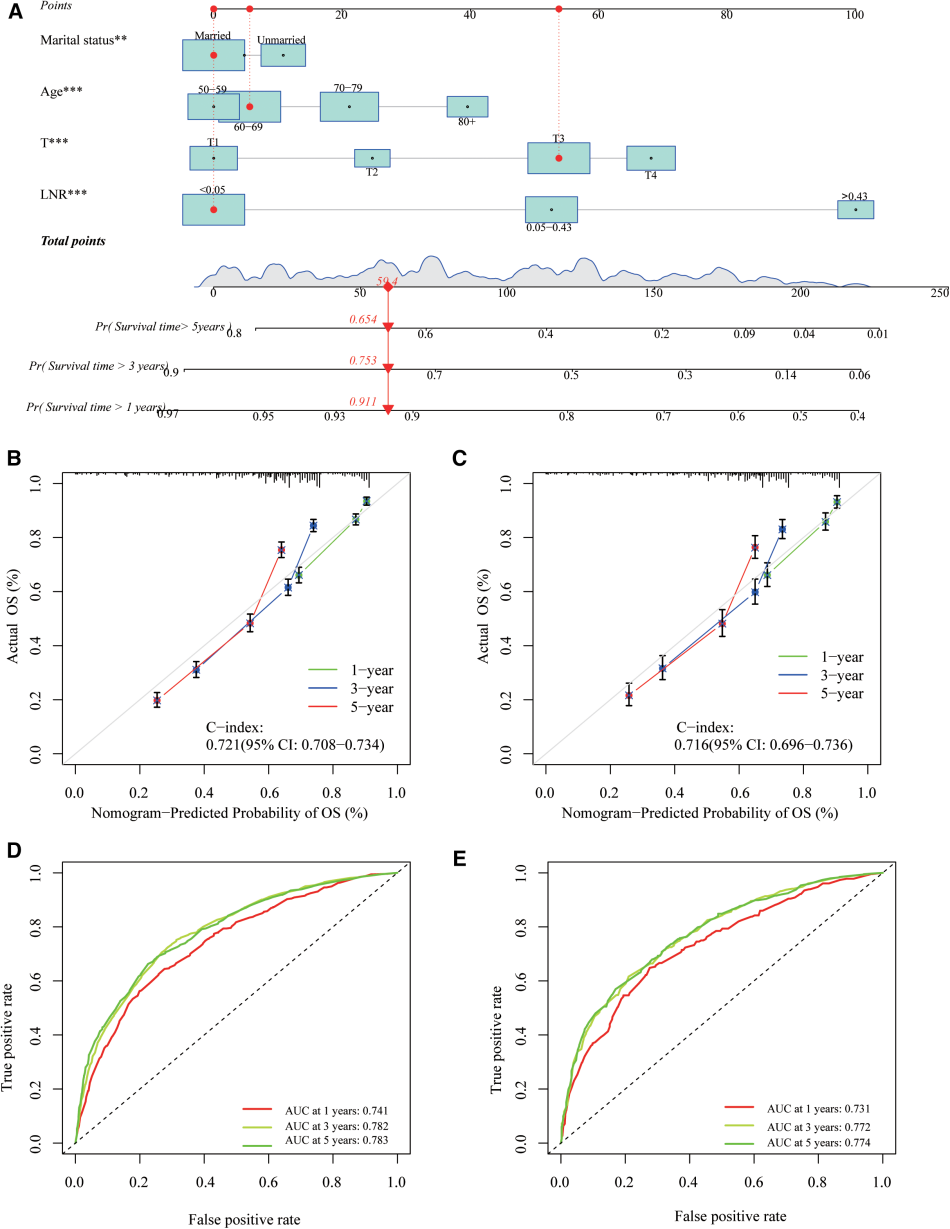
A nomogram based on LNR staging. (**A**) The nomogram was built based on four clinical variables in the training cohort. (**B,C**) The calibration curves and C-index values for predicting OS at 1-, 3-, and 5-year overall survival (OS) in the training and validation cohorts. (**D,E**) ROC curves showed good performance for predicting 1-, 3-, and 5-year OS in training and validation cohorts.

**Figure 6:**
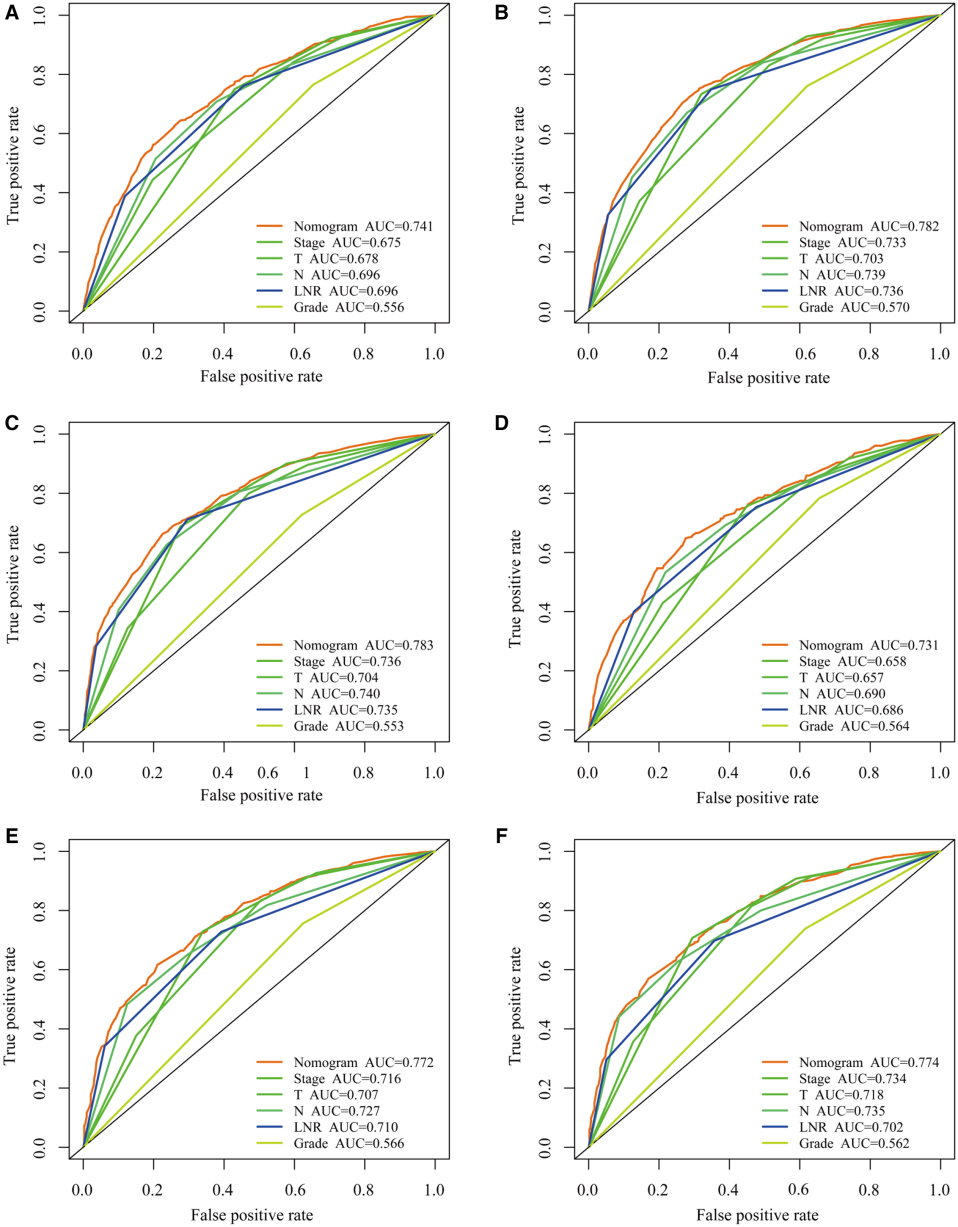
Comparing the predictive performance of nomogram with other clinical factors. (**A–C**) ROC curves for predicting 1-, 3-, and 5-year overall survival (OS) in the training cohort. (**D–F**) ROC curves for predicting 1-, 3-, and 5-year OS in the validation cohort.

**Figure 7:**
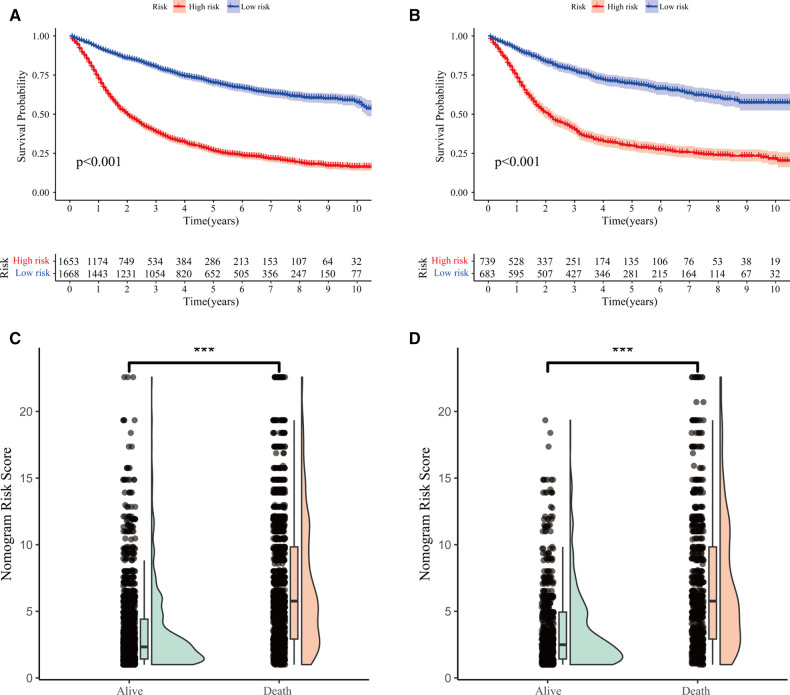
Predictive performance of nomogram. (**A,B**) Kaplan–Meier survival curves of LOGC patients with high and low risk in the training and validation cohorts, respectively. (**C,D**) Distribution of risk score and survival status of LOGC patients in the training and validation cohorts, respectively.

The LNR was selected as the optimal LN staging system to construct a novel nomogram for estimating the outcome of patients with LOGC (Figure 5A). Other predictive variables, including sex, marital status, chemotherapy, and T stage, were included. In the nomogram, each variable has a vertical scale line representing its range of values. By aligning the values of these variables and observing the intersection points on the nomogram, we can determine the estimated values of the 1-year, 3-year, and 5-year survival probabilities. Then, the calibration curves, ROC curves, DCA curves, and time-dependent C-index curves were plotted to evaluate the prediction performance of the nomogram (Figures 5B–E and Supplementary Figure S1), which suggested that the nomogram had good applicability and accuracy. The AUC of the nomogram in the training set was superior to that of the other variables at 1 year (AUC = 0.741), 3 years (AUC = 0.782), and 5 years (AUC = 0.783) and at 1 year (AUC = 0.731), 3 years (AUC = 0.772), and 5 years (AUC = 0.774) in the validation set (Figures 6D–F). The C-index of the nomogram was 0.721, which was greater than those of the LNR (C-index = 0.679) and tumor stage (C-index = 0.667) (Figures 6A–C). Moreover, according to the median risk score calculated from the nomogram, the patients in the training set were divided into high-risk and low-risk groups. The patients with a high nomogram risk score had a shorter survival time than those with a low nomogram risk score (Figure 7A). The nomogram risk scores of patients with different survival statuses are presented in Figures 7C–D. This indicated that as the nomogram risk score increased, the mortality of LOGC patients increased. Next, the risk scores were acquired from the same formula used for this nomogram. The patients in the validation set with low nomogram risk scores had better outcomes than the patients with high nomogram risk scores (Figure 7B). These results suggested that this nomogram could accurately and conveniently predict the prognosis of LOGC patients.

## Discussion

Over the past few decades, there has been a consistent decline in the occurrence of GC among middle-aged and elderly individuals (35, 36). However, compared with EOGC patients, LOGC patients presented significantly poor outcomes, especially those who underwent curative surgery (37). Therefore, it is necessary to pay more attention to the prognosis of patients with LOGC. Accurate survival prediction for LOGC patients is essential for determining their prognosis and making individualized treatment decisions. In this study, we explored the relationship between clinical features and the outcome of LOGC patients and confirmed the optimal LN staging system for patients with LOGC from the SEER database. This is the first study to identify a suitable LN system for LOGC patients via machine learning analysis and construct a nomogram for prognosis prediction.

LNM is a pivotal prognostic factor in GC patients (38, 39). Precise LN staging plays a critical role in treatment strategy selection and accurate prognosis prediction in cancer patients. The LNR and LODDS are alternative methods used to assess LN involvement in GC, refine the staging system, and provide more accurate prognostic information (40–42). The metastatic LNR was introduced in 2002 as a substitution method to initially forecast the prognosis of GC patients (43). We revealed the correlations between the LODDS, LNR, and pN stage and OS among patients with LOGC in the SEER database. The prediction abilities of the three LN systems, namely, LNR, LODDS, and pN, were compared via the AUCs, AICs, BICs, and C-indexes. However, there were fewer differences between them. Considering this situation, three machine learning methods, namely, LASSO, Xgboost, and RF analyses, were used to select the most important feature as the optimal LN system. Compared to the LODDS and pN stage, the LNR had a better ability to predict prognosis in LOGC patients when the total number of nodes examined was no less than 15, and the LNR was defined as the appropriate LN system in our study.

Sufficient perioperative LN retrieval is essential for the precise assessment of LN status (44, 45). Currently, three guidelines, namely, the Eighth Edition AJCC Cancer Staging Manual (46), the Chinese Society of Clinical Oncology (47), and the National Comprehensive Cancer Network (48), for GC patient management and treatment strategies recommend that no less than 15 LNs be acquired during surgery. With the increase in surgical management of GC, the proportion of patients with inadequate LNs will gradually decrease. Considering that inadequate LNs may misguide the judgment of the prognosis, only patients who had >16 LNs retrieved were included in our study.

In this study, we explored whether the pN stage, LNR, LODDS, T stage, marital status, and age at diagnosis were found to be independent prognostic factors for LOGC outcomes via multivariate regression. These results were similar to those of previous studies (49, 50). Marital status, an infrequently considered variable in GC research, exhibited a moderate impact on survival in our study. We found that patients who were married or ever married had better outcomes than those who were unmarried. This may be because married individuals possess higher subjective health perceptions, encounter fewer mental and physical health ailments, and exhibit extended life expectancy (51, 52).

Moreover, a promising nomogram was constructed to predict OS for LOGC patients based on the optimal LN system. Three variables, namely, marital status, age at diagnosis, and T stage, were also incorporated into the nomogram. The LOGC patients were assigned to high- or low-risk groups according to the nomogram. The survival rate analysis revealed that patients with higher risk scores had shorter survival times. The nomogram that we constructed demonstrated notably enhanced risk stratification abilities for LOGC patients compared to the stage from the AJCC eighth edition via ROC and DCA analyses. It can effectively aid in patient consultations regarding survival information, offering valuable guidance for clinical decision-making and the allocation of appropriate treatments. Despite the absence of a currently established optimal threshold for the LNR, it has been demonstrated to be the most reliable LN staging system. As attention to the LNR continues to grow, there is a prevailing belief that it will gain widespread acknowledgment in clinical settings in the foreseeable future.

In our study, we showed that the LNR was a more appropriate LN system for assessing patient prognosis. Despite similar findings reported in prior studies (53–59), some of which also utilized data from the SEER database (54, 58), our study possesses some distinct characteristics that differentiate it from earlier research. We collected SEER data from 2010 to 2020. Moreover, only patients who underwent curative surgery and had >16 LNs retrieved were selected, and follow-up analyses were conducted. This could partially explain the variance in the definition of the optimal LN system between this study and Che's study (60) and Aurello's study (61), in which LODDS was regarded as the best. The LODDS in these cases with a total number of nodes examined less than 16, which is not a minimum percentage, may hold greater importance than the LNR. Another peculiarity of our study involves the exclusive enrollment of GC patients who were diagnosed at over 50 years of age. Most middle-aged and elderly patients are diagnosed with stomach cancer (1). With the dramatic increase in the aging population, the average age of GC patients has increased at the same time (62). Finally, there are several limitations to this study. First, the study design employed here is retrospective and relies on data obtained from the SEER database, which may introduce some inherent bias. Some information, such as the location of metastatic LNs, was not recorded. Patients with metastatic LNs in the 8p, 12b/p, and 13 anatomical locations had a poorer prognosis than those without metastasis (63). Second, most of the patients in this study were white, and more extensive research involving diverse populations is necessary to corroborate and strengthen these findings.

## Conclusion

The LNR demonstrated a more powerful performance than other LN staging systems in LOGC patients after surgery. Our novel nomogram has better predictive accuracy in both the training and validation cohorts, which may aid in patient clinical decision-making.

## Data Availability

The raw data supporting the conclusions of this article will be made available by the authors, without undue reservation.

## Supplementary material

Supplementary Figure S1

Predictive performance of nomogram (**A,B**) The DCA curves for predicting OS in training and validation cohorts. (**C,D**) The time C-index curves of nomogram to predict the OS of LOGC patients in training and validation cohorts.
