# Supplementary figures and images for: Comparison of the predictive performance of three lymph node staging systems for late-onset gastric cancer patients after surgery
Source: Front Surg. 2024 Jun 11;11:1376702. doi: 10.3389/fsurg.2024.1376702 (PMC11196640; doi:10.3389/fsurg.2024.1376702)

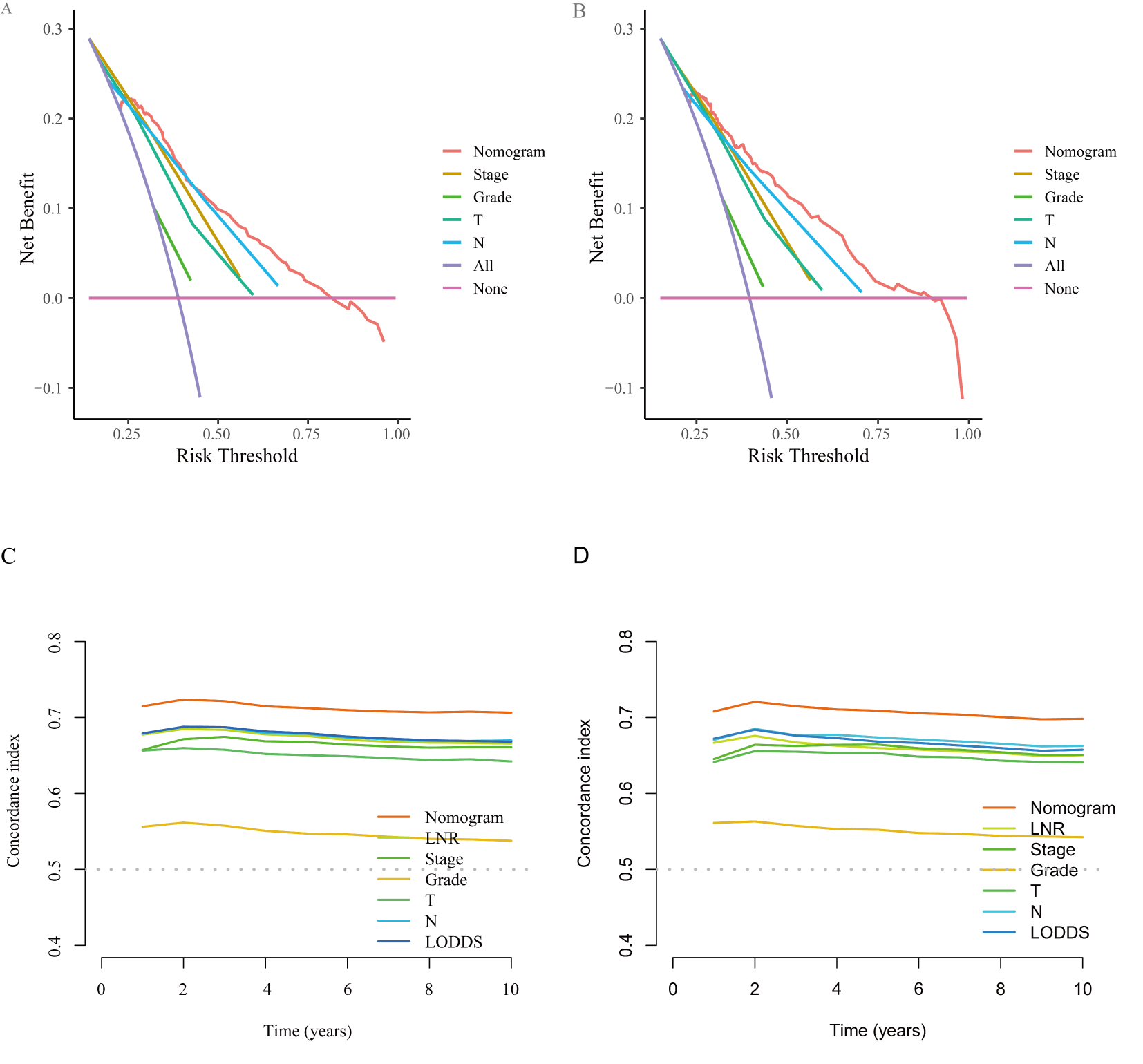

Supplement: Supplementary file 1 [file Image1.tif]
